# Choroidal Thickness in Thyroid Eye Disease: Comparison With Controls and Application in Diagnosing Non-Inflammatory Active Disease

**DOI:** 10.7759/cureus.19779

**Published:** 2021-11-20

**Authors:** Tarjani V Dave, Ramya Natarajan, Rakshi Ugandhar Reddy, Anasua G Kapoor, Vivek P Dave

**Affiliations:** 1 Oculoplastic Surgery, LV Prasad Eye Institute, Hyderabad, IND; 2 Ophthalmic Biophysics, LV Prasad Eye Institute, Hyderabad, IND; 3 Smt. Kanuri Santhamma Center for Vitreoretinal Diseases, LV Prasad Eye Institute, Hyderabad, IND; 4 Ophthalmic Plastic Surgery and Ocular Oncology Services, LV Prasad Eye Institute, Vijayawada, IND

**Keywords:** normal controls, active thyroid eye disease, non-inflammatory thyroid eye disease, thyroid eye disease, choroidal thickness

## Abstract

Introduction

Choroidal thickness is known to vary in various systemic diseases. In the current study, we aim to report the differences in choroidal thickness in thyroid eye disease (TED) and normals and its discriminatory value for differentiating various stages of TED.

Methods

Prospective, cross-sectional, non-interventional imaging study. In an institutional practice, 102 eyes of 51 patients were included and divided into five groups: normal controls (C), inactive TED (I), active TED (A), non-inflammatory active TED (NIA) and systemic thyroid disorder but no TED (SYS). Choroidal images were acquired using the swept-source optical coherence tomography (Topcon DRI OCT Triton) with automatic layer segmentation which provided an automatic measurement of the subfoveal choroidal thickness and the mean in nine subfields based on the Early Treatment Diabetic Retinopathy Study (ETDRS) grid. One-way analysis of variance (ANOVA), Youden index and area under the receiver operating characteristic curves (AUROC) were reported.

Results

Central choroidal thickness in the A group was 279±37.52 microns and in the NIA group was 302.5±59.22 microns. Both were comparable to each other and significantly higher than the C, I and SYS groups (p<0.001). All ETDRS sub-fields showed significant AUROC to distinguish NIA from I. Most significant Youden index was for the inner nasal and central ETDRS subfields (0.55 and 0.61 respectively). Inner nasal sub-field showed 100% specificity while the central sub-field, showed 86.5% for predicting NIA. At a choroidal thickness of >266 microns, the central sub-field had the strongest discriminatory potential to predict NIA.

Conclusion

Choroidal thickness is greater in active and non-inflammatory active TED. The inner nasal and central ETDRS sub-fields have value in differentiating the non-inflammatory active TED eyes from the inactive eyes.

## Introduction

Thyroid eye disease (TED) is an auto-immune disorder characterized by inflammation and cellular infiltrate into the orbital tissues. This results in an increase in orbital volume and resultant increase in intraorbital pressure [1,2]. Clinically, the disease activity and treatment approach are assessed most commonly by applying the clinical activity score (CAS) or the VISA score [3,4]. It is known, however, that the retinal structure and function in TED can get affected even when clinical activity is unapparent. It has been shown that a decrease in peripapillary and macular vascular density and increase in the retinal nerve fiber thickness are seen in active TED compared to inactive TED and healthy eyes. In disease inactivity, these parameters are comparable to healthy eyes [5].

Studies on choroidal thickness have been reported in systemic diseases including thyroid eye disease [5-7]. It is well known that the choroid receives > 70% of the ocular blood flow, and that the choroidal thickness may change in several inflammatory diseases [8]. Alterations in the ocular blood flow have been shown in patients with TED [8-10]. While the prevailing evidence points towards an overall increase in the choroidal thickness in active TED, the changes with respect to different regions of the choroid in active, inactive and control eyes are not known. Presently, there is also a lacuna regarding the predictive role of the choroidal thickness in picking up sub-clinical active TED. In the current communication, we report the comparative choroidal thickness in our subset of active TED, non-inflammatory active TED, inactive TED and normal control eyes over different areas of the macula. We also report the optimal choroidal thickness cut-off criteria at different regions in the macula with their respective sensitivities and specificities to pick up non-inflammatory active TED.

## Materials and methods

This was a prospective, cross-sectional, non-interventional imaging study conducted at a tertiary eye care center. Patients with TED presenting to the oculoplasty clinic of our center between September 2020 and March 2021 were included. Written informed consent was taken from all patients. The study was approved by the LV Prasad Eye Institute Ethics Committee (approval number LEC BHR-R-05-21-639) and adhered to all the Tenets of the Declaration of Helsinki. At the time of presentation, patients were identified as active (time since onset < 3 years) or inactive (time since onset > 3 years) [11]. Severity of inflammation and congestion were evaluated using the Clinical Activity Score (CAS) and the VISA inflammatory score [12]. A CAS score of ≥3/7 or VISA score of >4/10 was classified as clinically active disease. Simultaneously, normal healthy subjects with no history of ocular or systemic disorder and with no history of ocular surgery or treatment were included as controls. Patients were finally categorized into 5 groups - normal controls (C), inactive TED (I), active TED (A), non-inflammatory active TED (NIA) and those with systemic thyroid imbalance without TED (SYS). For all recruits, refractive error margins were set to the following spherical equivalent (SE) -4 D < SE < +4 D [13]. All choroidal thickness measurements were performed between 1 PM and 3 PM to limit the effects of the circadian rhythm [8].

Image acquisition protocol

**Figure 1:**
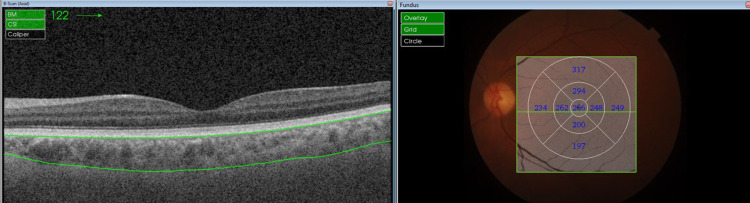
Choroidal thickness delineated along with thicknesses in the respective ETDRS sub-fields in inactive TED. ETDRS: Early Treatment Diabetic Retinopathy Study; TED: thyroid eye disease.

**Figure 2:**
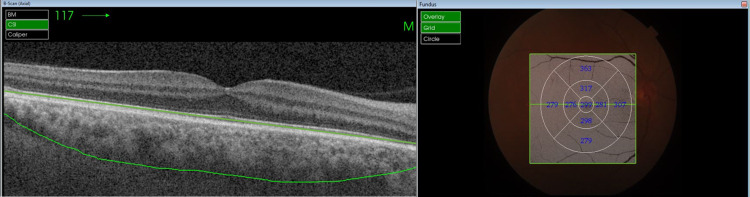
Choroidal thickness delineated along with thicknesses in the respective ETDRS sub-fields in active TED. ETDRS: Early Treatment Diabetic Retinopathy Study; TED: thyroid eye disease.

Choroidal images were acquired using the swept-source optical coherence tomography (Topcon DRI OCT Triton) with the following imaging specifications: 1050-μm wavelength, imaging speed of 100,000 A scans per second, axial resolution of 2.3 μm, and transverse resolution of 20 μm. We acquired 12 radial B-scans (12 mm long) and a volume of 512 B-scans using the 9 × 9-mm OCT-A mode, both centered on the fovea. Automatic layer segmentation using Topcon Advanced Boundary Segmentation software was verified by two observers (RUR and RN) and manually corrected when necessary by one experienced retina specialist (VPD) for each of the 12-mm radial scans to determine choroidal boundaries (inner boundary: Bruch’s membrane; outer boundary: choroid-scleral interface [CSI]) (Figures 1, 2).

Both observers had to agree on the need for manual correction modifying any of the original segmentations. The needful was done if deemed appropriate by both. Choroidal thickness (CT) measurements and CT maps were obtained from the 12 × 12-mm radial scans. CT maps derived from the 9 × 9-mm OCTA scans were based on an automatic segmentation using the manufacturer’s software. Subjects with poor OCT image quality were excluded.

The choroidal map obtained from the 12-mm radial scans provided an automatic measurement of the subfoveal CT (central 1 mm) and the mean CT in nine subfields based on the Early Treatment Diabetic Retinopathy Study (ETDRS) grid. The inner ring (3-mm diameter) included the superior inner macula, temporal inner macula, inferior inner macula, and nasal inner macula. The outer ring (6-mm diameter) was comprised of the superior outer macula, temporal outer macula, inferior outer macula, and nasal outer macula. The mean and SD and the coefficient of variation (CV) of CT in each macular subfield (= SD/mean) were calculated.

Statistical analysis

The data collected were arranged on an Excel spreadsheet and analyzed using the MedCalc Statistical Software version 19.7.2 (MedCalc, Ostend, Belgium). Continuous parametric data were compared using the independent sample t-test. Continuous non-parametric data were analyzed using the Wilcoxon rank-sum test. Appropriate 95% confidence intervals were calculated and reported for all measurements. One-way analysis of variance (ANOVA) was done to compare the means of the various choroidal thickness measurements between all the five groups simultaneously. Post-hoc testing for pair-wise comparison of the sub-groups where ANOVA was positive was done by the Student-Newman-Keuls test. Area under the receiver operating characteristic curves (AUROC) were calculated for the choroidal thickness values to determine the sensitivity and specificity in distinguishing inactive from NIA TED. The Youden index and the predictor cut-off value were also reported. Correlation coefficient was calculated to assess the correlation between the choroidal thickness recorded and the CAS scoring of the patients. A statistical difference of p<0.05 was assigned as significant.

## Results

Patient characteristics

One hundred and eighteen eyes of 59 patients were screened for the study of which 16 images of eight patients were of poor quality and were excluded. Finally, 102 eyes of 51 patients were included in the study. The patients were divided into five groups: normal controls (C), inactive TED (I), active TED (A), non-inflammatory active TED (NIA) and those with systemic thyroid disorder but no TED (SYS). The overall mean age was 44.52±10.02 years (median 46 years, range 19-65 years). The mean IOP was 16.1±3.37 mm Hg (median 16 mmHg, range 16-24 mmHg). The mean SE was -0.08±1.86 diopters (median 0, range -2.5 to +2.25). The best corrected visual acuities in all N and SYS cases were 20/20. One case each in the I group and NIA had BCVA of 20/40 whereas one case in the A group had a BCVA of 20/100.

Quantitative analysis of choroidal thickness

 Manual correction on the 12-mm radial scans was deemed necessary in 70% of the cases for the CSI and 10% cases for the inner margin of the choroid (Bruchs-RPE complex). The central subfoveal choroidal thickness in the A group was 279±37.52 microns and in the NIA group was 302.5±59.22 microns (Table 1).

**Table 1:** Choroidal thicknesses in the different ETDRS subfields with baseline demography, denoted as mean±standard deviation (median). ETDRS: Early Treatment Diabetic Retinopathy Study.

	Normal controls	Inactive TED	Active TED	Non-inflammatory active TED	Systemic thyroid disease but no eye disease
Number of eyes	24	28	18	20	12
Mean intraocular pressure	13.25±2.72 (14.5)	16±1.91 (16)	18.55±3.43 (20)	18.55±2.66 (18)	16.8±2.61 (16.5)
Sphero-equivalent	0.34±2.98 (0)	-0.36±0.57 (0)	-0.57±2 (0)	0.18±1.29 (0)	0.06±1.39 (-0.06)
Male gender	16 (67%)	16 (57%)	14 (77.8%)	14 (70%)	4 (33%)
Mean age (median)	42.75±4.55 (44)	42.85±10.84 (43.5)	46.22±12.10 (47)	43±11.46 (43)	52±7.28 (51)
Choroidal thickness in different regions					
Outer nasal	205.95±67.79 (219.5)	223±53.35 (227)	265.27±28.22 (265)	276.65±50.58 (292)	213±48.89 (206)
Inner nasal	217.79±53.21 (221.5)	240.92±42.02 (243)	283.66±30.06 (279)	309.25±60.36 (327)	234.25±45.97 (237)
Outer superior	208.29±34.01 (210.5)	254.6±42.07 (257)	278.61+56.71 (264)	273.2±56.1 (275.5)	208.33±37.64 (209)
Inner superior	217.75±44.52 (213)	248.89±53.84 (243.5)	277.5±45.42 (272)	288.9±56.09 (269.5)	212±39.48 (211.5)
Outer inferior	218.04±46.04 (211)	225.25±54.78 (219)	277.88±37.49 (285)	257.35±68.22 (268)	204.25±57.74 (205)
Inner inferior	221.33±45.72 (228.5)	236.35±44.65 (231)	272.27±27.11 (272)	273.15±78.58 (283.5)	203.58±36.36 (208.5)
Outer temporal	200.16±51.02 (199)	209.85±48.35 (208.5)	254.33±34.51 (255)	237.8±54.98 (241.5)	218.16±35.57 (223)
Inner temporal	221.12±51.06 (219)	232.78±52.07 (242)	263.05±33.52 (270.5)	263.65±59.64 (281)	214.83±46.13 (211.5)
Central	219.25±42.9 (226)	230.67±44.5 (233)	279±37.52 (289)	302.5±59.22 (317)	225.83±27.56 (217)

**Figure 3:**
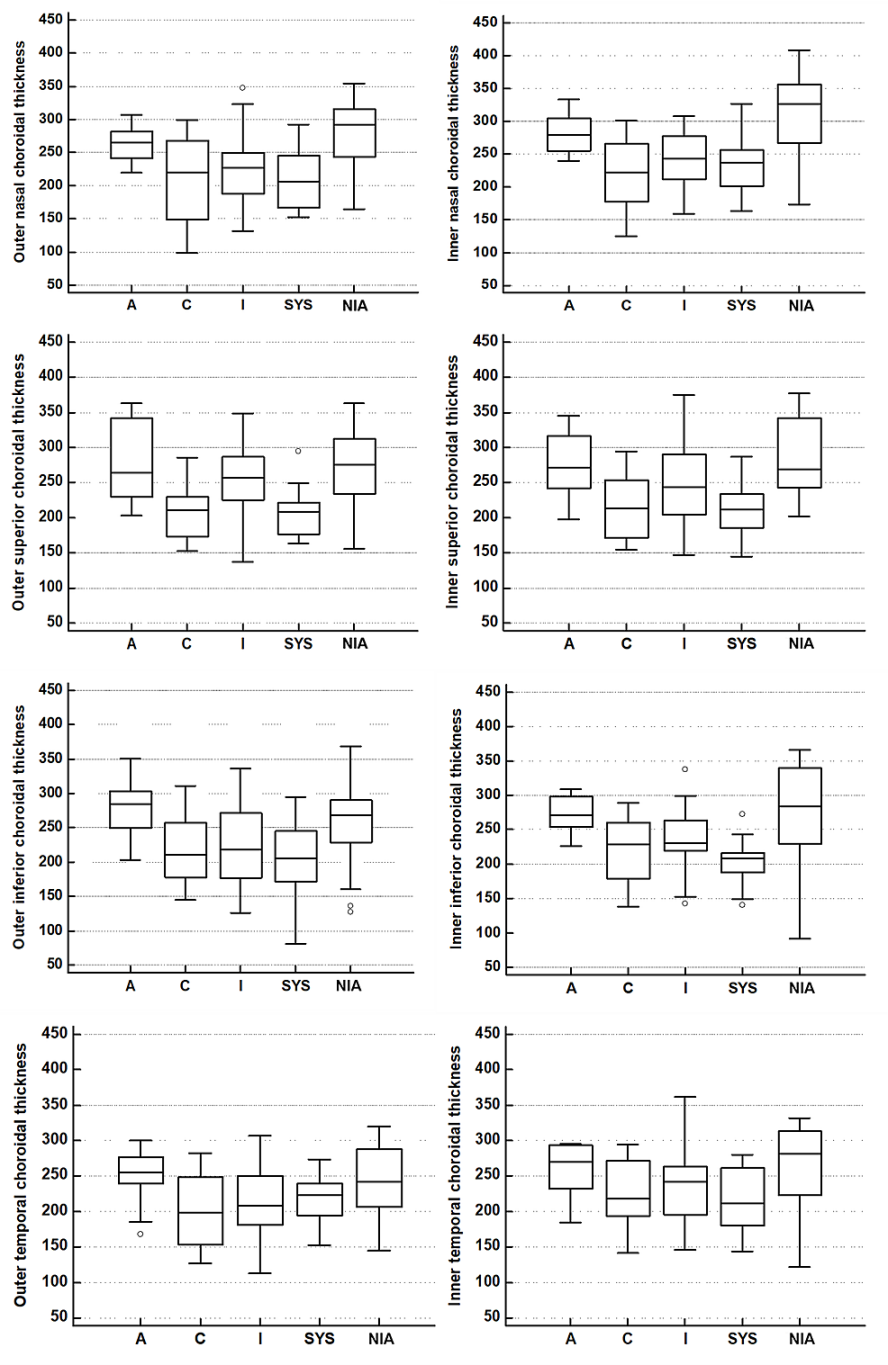
Bar graphs showing comparative choroidal thicknesses in the various ETDRS sub-fields across the various groups: active (A), controls (C), inactive TED (I), systemic thyroid disorder without TED (SYS) and non-inflammatory active TED (NIA). ETDRS: Early Treatment Diabetic Retinopathy Study; TED: thyroid eye disease.

Both of these were comparable to each other but were significantly higher than the C, I and SYS groups (p<0.001). On similar lines for most of the ETDRS sub-fields especially the nasal ones, the thicknesses in the A and NIA group were comparable but significantly higher than the C, I and SYS groups (Figure 3).

Statistically, the highest f-ratio for ANOVA was noted in the inner nasal and the central ETDRS sub-field.

**Figure 4:**
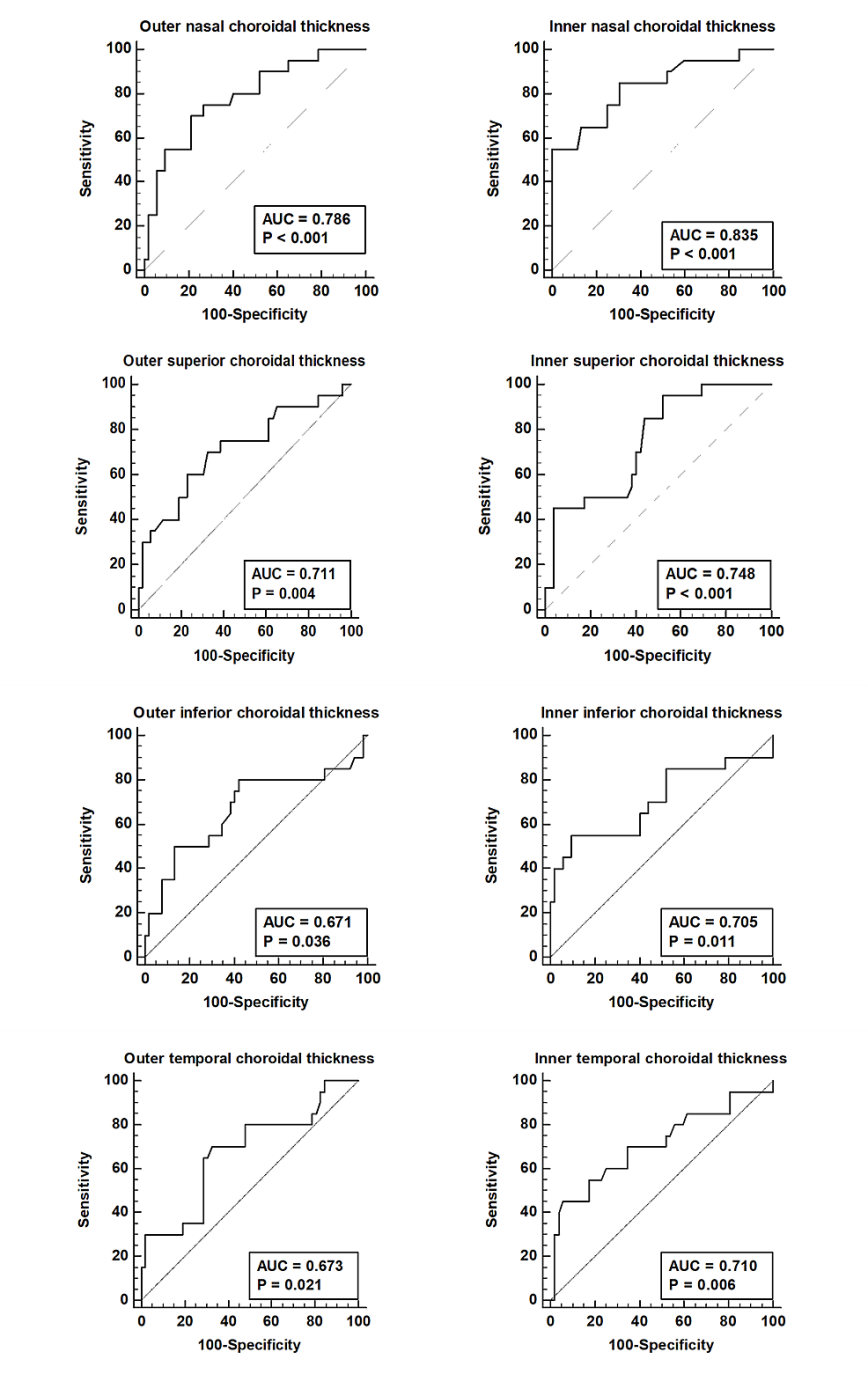
Graph showing comparative AUROC in the various ETDRS sub-fields across the various groups: active (A), controls (C), inactive TED (I), systemic thyroid disorder without TED (SYS) and non-inflammatory active TED (NIA) ETDRS: Early Treatment Diabetic Retinopathy Study; TED: thyroid eye disease; AUROC: area under the receiver operating characteristic curves.

**Figure 5:**
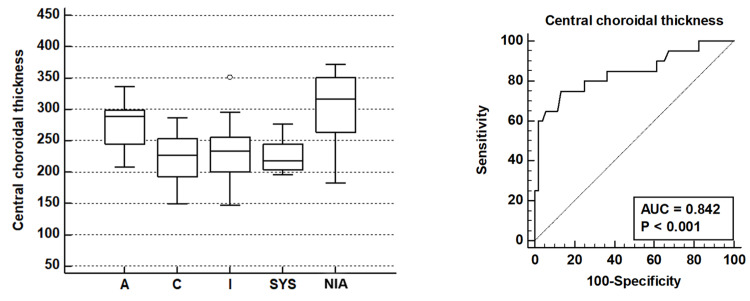
Bar graph for choroidal thickness and AUROC for the central ETDRS sub-field. ETDRS: Early Treatment Diabetic Retinopathy Study; AUROC: area under the receiver operating characteristic curves.

 All ETDRS sub-fields showed statistically significant AUROC values (Figures 4, 5).

The most significant Youden index was noted for the inner nasal and the central ETDRS subfields (0.55 and 0.61, respectively). The inner nasal sub-field showed a specificity of 100% while the central sub-field showed a specificity of 86.5% for predicting active disease when clinical signs were lacking. At a choroidal thickness value of >226 microns, the central sub-field had the strongest discriminatory potential to predict active TED with a sensitivity of 75% and a specificity of 86.5%.

For the A, I and NIA groups CAS scores were noted. Correlation coefficient was calculated to assess the correlation between the choroidal thickness recorded and the CAS. For most of the ETDRS sub-fields, there was a statistically significant correlation of the choroidal thickness with the CAS for the I and the A groups. For the NIA group; however, none of the sub-fields showed any statistical correlation with the CAS (Table 2).

**Table 2:** Correlation of clinical activity score (CAS) and the choroidal thickness in inactive, active and non-inflammatory active TED in the different ETDRS sub-fields. Values are represented as mean±standard deviation. Parenthesis represents range of 95% confidence intervals. ETDRS: Early Treatment Diabetic Retinopathy Study; TED: thyroid eye disease.

	Mean choroidal thickness in inactive and active TED	Mean CAS score	Correlation coefficient (95% CI range)	p- value	Mean choroidal thickness in non-inflammatory active TED	Mean CAS score	Correlation coefficient	p-value
Outer nasal	239.54±49.44	1.95±1.31	0.41 (0.14 to 0.61)	0.004	276.65±50.58	0.85±1.09	0.31 (-0.14 to 0.67)	0.17
Inner nasal	257.65±42.96	1.95±1.31	0.42 (0.15 to 0.63)	0.004	309.25±60.36	0.85±1.09	0.19 (-0.26 to 0.58)	0.4
Outer superior	264±48.97	1.95±1.31	0.31 (0.25 to 0.55)	0.03	273.2±56.1	0.85±1.09	-0.16 (-0.56 to 0.3)	0.49
Inner superior	260.08±52.13	1.95±1.31	0.27 (-0.18 to 0.52)	0.06	288.9±56.09	0.85±1.09	-0.07 (-0.5 to 0.3)	0.74
Outer inferior	245.84±54.83	1.95±1.31	0.34 (0.52 to 0.55)	0.02	257.34±68.22	0.85±1.09	0.33 (-0.13 to 0.67)	0.15
Inner inferior	250.41±42.28	1.95±1.31	0.26 (-0.32 to 0.52)	0.08	273.15±78.58	0.85±1.09	0.12 (-0.33 to 0.53)	0.12
Outer temporal	227.26±48.31	1.95±1.31	0.31 (0.28 to 0.55)	0.03	237.8±54.98	0.85±1.09	0.26 (-0.2 to 0.63)	0.25
Inner temporal	244.63±47.69	1.95±1.31	0.28 (-0.14 to 0.52)	0.06	263.65±59.64	0.85±1.09	0.16 (-0.3 to 0.56)	0.49
Central	249.58±47.84	1.95±1.31	0.34 (0.04 to 0.57)	0.02	302.5±59.22	0.85±1.09	0.17 (-0.28 to 0.57)	0.44

Bivariate and multivariate regression analysis was run for the effect of age, gender, IOP, SE, exophthalmometer reading, CAS and the BCVA in the central and the inner nasal choroidal thicknesses (Table 3).

**Table 3:** Bivariate and multivariate regression analysis of various factors potentially affecting the choroidal thickness in the central and the inner nasal ETDRS sub-fields. CAS: clinical activity score; IOP: intraocular pressure; BCVA: best-corrected visual acuity; logMAR: logarithm of minimal angle of resolution; ETDRS: Early Treatment Diabetic Retinopathy Study; TED: thyroid eye disease. Values are represented as mean±standard error.

	Bivariate analysis		Multivariate analysis	
	Coefficient of regression±SE	p-value	Coefficient of regression±SE	p-value
Central choroidal thickness				
Age	-0.5±0.59	0.39	-0.8±0.7	0.04
Male gender	6.77±14.38	0.63	40.91±18.81	0.07
IOP	1.81±1.47	0.22	1.71±1.55	0.27
Exophthalmometer reading	0.98±2	0.62	4.1±2.43	0.1
CAS	11.92±5.17	0.03	11.89±6.76	0.08
Spheroequivalent	-3.35±5.19	0.52	-3.53±6.1	0.56
BCVA logMAR	4.61±3.94	0.24	1.09±4.32	0.8
Inner nasal choroidal thickness				
	Coefficient of regression±SE	p-value	Coefficient of regression±SE	p-value
Age	-0.59±0.57	0.3	-1.34±0.62	0.03
Male gender	-3.46±13.86	0.8	24.7±16.74	0.14
IOP	0.49±1.43	0.73	-0.92±1.38	0.5
Exophthalmometer reading	0.55±1.93	0.77	1.2±2.16	0.58
CAS	13.17±4.5	0.005	16.5±6.01	0.009
Spheroequivalent	1.59±5.01	0.75	2.39±5.43	0.66
BCVA logMAR	2.8±3.82	0.46	1.4±3.84	0.71

The only factor that positively correlated to the increasing thickness was the CAS. Age correlated negatively while the rest of the clinical and demographic factors did not have any bearing on the choroidal thickness.

## Discussion

The current study describes the comparative choroidal thickness in the various ETDRS sub-fields in normal control eyes, eyes with active TED, inactive TED, non-inflammatory active TED and those in patients with systemic thyroid disorder but no TED. The study showed that choroidal thickness is significantly higher in active TED and non-inflammatory active TED as compared to the other groups. As the NIA eyes do not have any clinical signs of activity and have low CAS, the choroidal thickness, especially the inner nasal and the central sub-fields have a good value in differentiating the timeline-wise active eyes from the inactive eyes.

Caliskan et al first described the changes in choroidal thickness in eyes with TED [6]. They reported that the mean subfoveal choroidal thickness increases in eyes with active TED. They also reported thicknesses at 1.5 mm and 3 mm distances from the fovea, both nasal and temporal and reported them to be greater than corresponding thicknesses in normal control and inactive eyes. While in the current study, the thickness changes were greater in the nasal half, those in the study by Caliskan et al, were greater temporally. They proposed subfoveal choroidal thickness to be a useful parameter to monitor disease activity. In the current study, both subfoveal central choroidal thickness and inner nasal choroidal thickness were good predictors of activity even in the absence of clear clinical signs.

Lai and colleagues in 2019, described their findings of choroidal thickness in thyroid-associated orbitopathy (TAO) [14]. Akin to the current study, they noted a thicker choroid sub-foveally and a negative correlation of the choroidal thickness with age. They also noted a worsening of exophthalmos and poorer BCVA with a thinner choroid. Such an association was not seen in the current study. Yu et al, in their study, assessed the retinal and choroidal variations in thyroid-associated ophthalmopathy. They noted an increased choroidal and retinal nerve fiber layer thickness in eyes with active thyroid eye disease. The receiver operating characteristic (ROC) curve and the area under curve (AUC) were conducted to assess the diagnostic capability of different parameters in TAO. Comparisons of choroidal thickness yielded ROC curve areas of 0.814 for active TAO/normal and 0.828 for inactive TAO/normal (p < 0.001, p < 0.001). The AUC analysis indicated that the choroidal thickness exhibited a significant discriminatory power in TAO diagnosis. They also mention a limitation that, dysthyroid patients in the absence of TAO were not enrolled in their study. In the current study we noted a significant discriminatory power of the central and the inner nasal choroidal thickness according to the ETDRS grid for diagnosing active and non-inflammatory active TED. We also reported these measurements in dysthyroid patients in the absence of TED.

The Rundle’s curve has been used for many years as a paradigm to describe the natural history of TED [11,15,16]. According to Rundle’s curve, TED signs and symptoms worsen rapidly during an initial phase, up to a peak of maximum severity, and then improve. The signs and symptoms finally reach a static plateau without, however, resolving into normalcy. The common interpretation is that the curve of TED inflammatory signs and symptoms, is slightly separated from the curve of TED clinical severity, usually evaluated based on the degree of proptosis, eyelid aperture, diplopia, and visual acuity. According to this model, the activity peak would precede the severity peak by a few months. It is now known that not all cases of “active TED” are characterized by active measurable inflammatory signs, and that the relationship between inflammation and severity of outcome is not well established as shown in a series by Ugradar and colleagues [17]. Lim et al, in their study on TED in south-east Asian patients note that even patients with a new-onset TED, a VISA score of less than 4/10 and experiencing significant symptoms can be classified as active TED [18]. Along similar lines, we categorized one of the comparative groups in the present study as non-inflammatory active TED (NIA). In the current study, we found that the central subfoveal choroidal thickness in the active TED group and in the NIA group were comparable to each other but were significantly higher than the normal controls, inactive TED and the systemic thyroid disease groups. For the measured CAS in this study, there was a significant correlation of the choroidal thickness with the CAS for the inactive and the active TED groups. For the NIA group; however, none of the sub-fields showed any statistical correlation with the CAS. This corroborates the presence of a subset of active TED which is minimally/non-inflammatory (having low CAS) but is ideally classified overall as active (choroidal thickness parameters comparable to classical active TED cases).

The biggest strength of the current study is the prospective collection of data with well-defined groups. This is the first study of its kind to compare the choroidal thicknesses across normals and TED cases with those having only systemic thyroid disorder without TED. This also is the first novel description of the choroidal anatomic changes in NIA subset of TED. The weakness of the study is its cross-sectional study design which did not allow repeat temporal imaging of the same patient. This could have allowed for an opportunity to assess the changes in the choroid in those cases where disease activity changed over time.

## Conclusions

In conclusion, the current study showed that choroidal thickness is significantly higher in active TED and non-inflammatory active TED as compared to the other groups. As the NIA eyes do not have any clinical signs of activity and have low CAS, we propose that the choroidal thickness, especially the inner nasal and the central ETDRS sub-fields have a good value in differentiating the non-inflammatory active TED eyes from the inactive eyes.
